# Morphoproteomics and biomedical analytics coincide with clinical outcomes in supporting a constant but variable role for the mTOR pathway in the biology of congenital hyperinsulinism of infancy

**DOI:** 10.1186/s13023-017-0735-9

**Published:** 2017-12-16

**Authors:** Robert E. Brown, Senthil Senniappan, Khalid Hussain, Mary F. McGuire

**Affiliations:** 1 Department of Pathology and Laboratory Medicine, UT Health McGovern Medical School, Houston, TX USA; 2 Department of Paediatric Endocrinology, Alder Hey Children’s Hospital, Liverpool, L12 2AP UK; 3 Department of Paediatric Medicine Division of Endocrinology, Sidra Medical & Research Center OPC, C6-337 PO Box 26999, Doha, Qatar; 4 Biomedical Analytics, Houston, TX USA

**Keywords:** Morphoproteomics, Biomedical analytics, Congenital hyperinsulinism of infancy, Sirolimus

## Abstract

We first introduced the concept of the mTOR pathway’s involvement in congenital hyperinsulinism of infancy (CHI), based largely on morphoproteomic observations and clinical outcomes using sirolimus (rapamycin) as a therapeutic agent in infants refractory to octreotide and diazoxide treatment. Subsequent publications have verified the efficacy of such treatment in some cases but limited and variable in others. We present further evidence of a constant but variable role for the mTOR pathway in the biology of CHI and provide a strategy that allows for the short-term testing of sirolimus in individual CHI patients.

The severe form of diffuse hyperinsulinemic hypoglycemia is mainly associated with mutations in *ABCC8* and *KCNJ11* that are unresponsive to diazoxide and/or octreotide therapy [1, 2]. This poses a threat to the infants with CHI not only by causing potential neurological damage leading to epilepsy, cerebral palsy and adverse neurological development in up to 40% of cases [3, 4] but also necessitating in some, near total pancreatectomy. Furthermore, 59% of such surgically treated patients can show persistent hyperinsulinemic hypoglycemia for up to 5 years post-surgery, and eventually diabetes mellitus will be manifested in all by the time they reach early adolescence [5].

The microanatomical characteristics of the diffuse form of CHI include non-proliferative, islet cell nucleomegaly [6]. In this context we have shown that the mammalian target of rapamycin (mTOR) protein, phosphorylated (p) on serine 2448, p-mTOR (Ser 2448) is overexpressed but variably on the plasmalemmal aspect of the acinar cells in CHI [7] and variably in the cytoplasm and nuclei of the insulin-producing islet cells, including those with karyomegaly [8] (Fig.1).

**Fig. 1:**
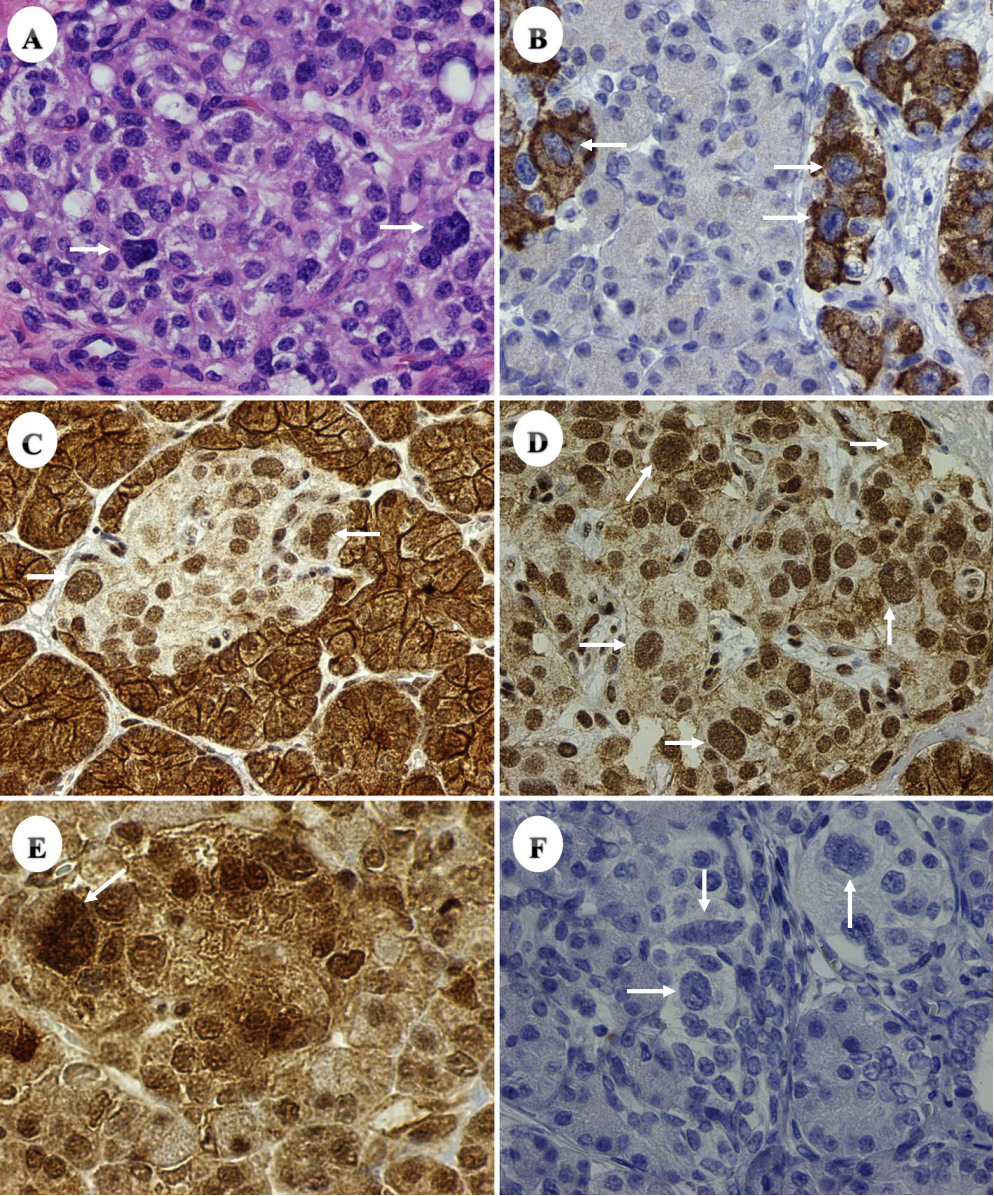
Pancreas of infant with CHI and paternal ABCC8 showing nucleomegaly (arrows) in islet cells, with insulin production (Frames **a**, H&E and **b**, beta cells with insulin), p-mTOR (Ser 2448) on the plasmalemmal aspect of the acinar cells and positivity in the islet cells with nucleomegaly (Frames **c** and **d**), p-Akt (Ser 473), expression in the islet cells with nucleomegaly (Frame **e**) and contrastively, the negative control (Frame **f**) (Original magnifications frames **a**-**d** and F ×400 and ×600 for frame **e**)

Biomedical analytics using Ingenuity Pathway Analysis and data mining of the National Library of Medicine’s Medline Data Base confirms the role of the mTOR pathway [8] in the biology of CHI with actionable therapeutic targets. A CHI network was constructed using the known CHI-associated genes described by Dunne and Banerjee’s associates [9]: *GLUD1, SLC16A1, HADH, UCP2, KCNJ11, ABCC8, HNF1A, GCK, HNF4A*.

The network showed that the MTOR-related molecules: MTOR, Raptor, Rictor, MTORC1 (but not MTORC2) interacted with all 9 CHI genes (Fig. 2). More than 300 interactions were identified (not shown due to complexity of image). Sirolimus modulated the MTOR group and thus, indirectly, the 9 CHI gene group. Sirolimus also increased expression of human UCP2 mRNA in the 9 CHI group [10].

**Fig. 2:**
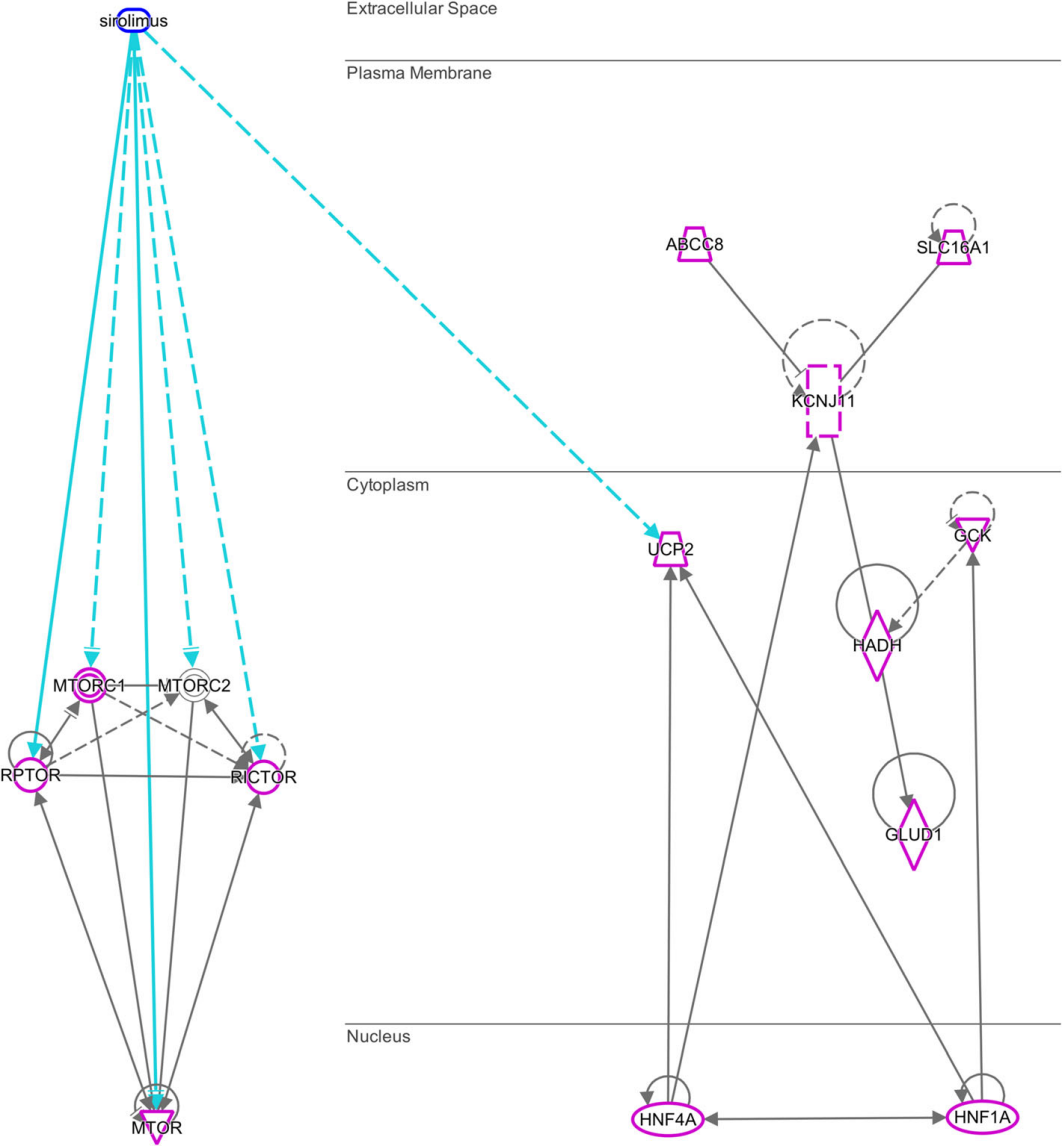
MTOR-related molecules (left) have more than 300 interactions (magenta highlights) with all 9 CHI genes (right) identified by Dunne and Banerjee [9] (interactions not shown due to density.) Direct interactions: solid lines; indirect interactions: dashed lines. Activation/expression: (−--➔), inhibition: (−--|), inhibits and acts upon: (−--|>)

Parenthetically, the contention by Banerjee and colleagues [4] that mTOR mRNA equates to *mTOR* gene in pancreases from normal, focal CHI, and diffuse CHIexpression could be accurate. Alternatively in the context of the morphoproteomic evidence of variable but constant activation and overexpression of the mTOR pathway in pancreases from diffuse CHI, it could represent the resultant of a steady state of transcription, translation and utilization of mTOR mRNA [11] following the integration of genomic, proteomic and pathway biology. Notably pathway ontology associated with the CHI disease network includes mTOR signaling [9]. Moreover, the clinical outcomes coincide with the findings of a variable therapeutic success in using sirolimus in the treatment of CHI [2, 12–18]. In the context of risk [3–5] versus benefit, we hold that severe diffuse CHI infants deserve a trial with an appropriate dose of sirolimus to see whether it is effective keeping in mind and monitoring for the potential immunosuppressive and adverse consequences of sirolimus [2]. We agree that sirolimus with or without other combinatorial therapies (octreotide, nifedipine, exendin-(9-39) and metformin) to counter the variable but constant activation of the mTOR biology in CHI should be explored in larger clinical trials.

## References

[1] Senniappan S, Shanti B, James C, Hussain K (2012). Hyperinsulinaemic hypoglycaemia: genetic mechanisms, diagnosis and management. J Inherit Metab Dis.

[2] Senniappan S, Alexandrescu S, Tatevian N, Shah P, Arya V, Flanagan S, Ellard S, Rampling D, Ashworth M, Brown RE, Hussain K (2014). Sirolimus therapy in infants with severe hyperinsulinemic hypoglycemia. N Engl J Med.

[3] Shah P, Demirbilek H, Hussain K (2014). Persistant hyperinsulinaemic hypoglycemia in infancy. Semin Pediatr Surg.

[4] Banerjee I, De Leon D, Dunne MJ (2017). Exreme caution on the use of sirolimus for the congenital hyperinsulinism in infancy patient. Orphanet J Rare Dis.

[5] Beltrand J, Caquard M, Arnoux JB, Laborde K, Velho G, Verkarre V, Rahier J, Brunelle F, Nihoul-Fekete C, Saudubray JM, Robert JJ, de Lonlay P (2012). Glucose metabolism in 105 children and adolescents after pancreatectomy for congenital hyperinsulinism. Diabetes Care.

[6] Han B, Newbould M, Batra G, Cheesman E, Craigie RJ, Mohamed Z, Rigby L, Padidela R, Skae M, Mironov A, Starborg T, Kadler KE, Cosgrove KE, Banerjee I, Dunne MJ (2016). Enhanced islet cell Nucleomegaly defines diffuse congenital Hyperinsulinism in infancy but not other forms of the disease. Am J Clin Pathol.

[7] Alexandrescu S, Tatevian N, Olutoye O, Brown RE (2010). Persistent hyperinsulinemic hypoglycemia of infancy: constitutive activation of the mTOR pathway with associated exocrine-islet transdifferentiation and therapeutic implications. Int J Clin Exp Pathol.

[8] Senniappan S, Brown RE, Hussain K (2016). Genomic and morphoproteomic correlates implicate the IGF-1/mTOR/Akt pathway in the pathogenesis of diffuse congenital hyperinsulinism. Int J Clin Exp Pathol.

[9] Stevens A, Cosgrove KE, Padidela R, Skae MS, Clayton PE, Banerjee I, Dunne MJ (2013). Can network biology unravel the aetiology of congenital hyperinsulinism?. Orphanet J Rare Dis.

[10] Peng T, Golub TR, Sabatini DM (2002). The immunosuppressant rapamycin mimics a starvation-like signal distinct from amino acid and glucose deprivation. Mol Cell Biol.

[11] Li JJ, Biggin MD (2015). Gene expression. Statistics requantitates the central dogma. Science.

[12] Shah P, Arya VB, Flanagan SE, Morgan K, Ellard S, Senniappan S, Hussain K (2015). Sirolimus therapy in a patient with severe hyperinsulinaemic hypoglycaemia due to a compound heterozygous ABCC8 gene mutation. J Pediatr Endocrinol Metab.

[13] Abraham MB, Shetty VB, Price G, Smith N, Md B, Siafarikas A, Resnick S, Whan E, Ellard S, Flanagan SE, Davis EA, Jones TW, Hussain K, Choong CS (2015). Efficacy and safety of sirolimus in a neonate with persistent hypoglycaemia following neartotal pancreatectomy for hyperinsulinaemic hypoglycaemia. J Pediatr Endocrinol Metab.

[14] Minute M, Patti G, Tornese G, Faleschini E, Zuiani C, Ventura A (2015). Sirolimus therapy in congenital Hyperinsulinism: a successful experience beyond infancy. Pediatrics.

[15] Meder U, Bokodi G, Balogh L, Korner A, Szabo M, Pruhova S, Szabo AJ (2015). Severe Hyperinsulinemic hypoglycemia in a neonate: response to Sirolimus therapy. Pediatrics.

[16] Guemes M, Shah P, Roženkova K, Gilbert C, Morgan K, Hussain K (2016). Severe Hyperinsulinaemic Hypoglycaemia in Beckwith-Wiedemann syndrome due to paternal Uniparental Disomy of 11p15.5 managed with Sirolimus therapy. Horm Res Paediatr.

[17] Unal S, Gonulal D, Ucakturk A, Siyah Bilgin B, Flanagan SE, Gurbuz F, Tayfun M, Elmaoğulları S, Araslı A, Demirel F, Ellard S, Hussain K (2016). A novel homozygous mutation in the KCNJ11 gene of a neonate with congenital Hyperinsulinism and successful management with Sirolimus. J Clin Res Pediatr Endocrinol.

[18] Szymanowski M, Estebanez MS, Padidela R, Han B, Mosinska K, Stevens A, Damaj L, Pihan-Le Bars F, Lascouts E, Reynaud R, Ferreira C, Bansept C, de Lonlay P, Saint-Martin C, Dunne MJ, Banerjee I, Arnoux JB (2016). Mammalian target of rapamycin (mTOR) inhibitors for the treatment of severe congenital Hyperinsulinism: perspectives on limited therapeutic success. J Clin Endocrinol Metab.

